# Insights from multidisciplinary rare disease visits: Findings from wrap-up documents and participant surveys in a national diagnostic study

**DOI:** 10.1016/j.rare.2025.100105

**Published:** 2025-09-13

**Authors:** Lindsay E. Rosenfeld, Kimberly LeBlanc, Anna Nagy, Jorick Bater, Undiagnosed Diseases Network, Alexa T. McCray

**Affiliations:** a Boston University School of Social Work, Institute for Equity in Child Opportunity and Healthy Development, United States; b Harvard T.H. Chan School of Public Health, Department of Social and Behavioral Sciences, United States; c Harvard Medical School, Department of Biomedical Informatics, United States; d Mass General Brigham, Partners Personalized Medicine, United States; e NYU Langone, Department of Epidemiology, United States; f Division of Clinical Informatics, Beth Israel Deaconess Medical Center, United States

**Keywords:** Rare disease, Care recommendations, Diagnostic odyssey

## Abstract

**Background:**

This study is part of the Undiagnosed Diseases Network (UDN). The UDN is designed to improve diagnostic evaluation for patients who have defied diagnosis as well as to identify the underlying mechanism of disease. We explored recommendations made as part of the UDN evaluation, the number and type of specialists involved in that evaluation, and factors related to changes in care following the UDN visit. We used two datasets: visit wrap-up documents and post-evaluation surveys. We created distinct statistical models for each dataset to further understand the patient experience after a multidisciplinary rare disease visit.

**Results:**

Across datasets, the most common primary symptom category was neurology. The mean number of evaluations per participant was 3.8 with the most common being Genetics. The mean number of recommendations per participant was 4.9; 81.5 % had ≥ 1 recommendation. The most common recommendation was referral to a medical provider. Having a diagnosis within 3 months of the UDN visit was associated with more evaluations, but fewer recommendations.

**Conclusion:**

UDN visits typically involve evaluations by multiple specialists, potentially resulting in recommendations for downstream care. In the survey sample, over half the participants cited their intention to follow the recommendations made. Maximizing communication about ongoing care management following the UDN visit may also optimize care. Future research can explore how and why such characteristics matter.

## Background

For patients and families affected by rare conditions, the journey to identify the cause of disease can involve years of medical evaluation and intervention, with the average length of time to diagnosis being over 6 years [1]. For patients and families, the ultimate goal for most is to find an accurate diagnosis to explain symptoms, end uncertainty, and plan a path forward. For many, a certain diagnosis is not yet attainable [2-5]. Recently, genomic testing has emerged as a powerful tool in the diagnostic process, helping to narrow down, and often identify, the cause of rare conditions [6-8].

Results from numerous research studies support the benefits of genomic testing in rare disease contexts for both the patient and caregiver. For example, patients and caregivers have expressed benefits to quality of life, medical care, uncertainty, worry, and personal utility following diagnostic testing [9-12]. Personal utility is a complex construct encompassing medical management (e.g., clinician-directed activities) and affective (e.g., enhanced coping, mental preparation, feeling of responsibility, improved spiritual wellbeing), cognitive (e.g., value of information, knowledge of the condition, curiosity, self-knowledge), behavioral (e.g., ability for future planning, reproductive autonomy, communication), and social (e.g., concern over discrimination, feeling good for helping others, change in social support) domains [11]. However, few studies have focused on what occurs after genomic testing (e.g., increase in services, care management), and genome sequencing in particular, for rare conditions. For example, we do not fully understand possible subsequent changes to referrals, treatment, and management, especially in adult populations, as such insurance coverage for sequencing varies [13-15].

The Undiagnosed Diseases Network (UDN), a research study funded by the National Institutes of Health (2013-present), was developed to diagnose participants who have defied diagnosis as well as to identify the underlying mechanism of disease. To be eligible, participants must have one or more objective findings pertinent to the phenotype for which a UDN application was submitted and no diagnosis despite evaluation by at least two specialists who assessed the patient for the objective finding(s). Patients apply directly to the UDN and need to supply a recommendation letter from a health care provider. UDN visits involve genome sequencing as well as careful examination by multiple experts using cutting-edge technologies [4,16,17]. Data generated through evaluation visits that use this information are shared in a central database by site personnel. Post-evaluation surveys provide further insight into the experience. The network’s unique resources have enabled its diagnostic success, facilitating diagnosis for a third of patients [4]. It is clear the network adds value in terms of ability to diagnose patients who have exhausted clinical options [18-21]. However, there is little evidence about what happens when genomic testing and extensive clinical workups are combined [22].

We explored 1) recommendations made as part of the UDN evaluation as well as the number and type of specialists involved in that evaluation and 2) factors that led to UDN recommendations for changes in care following the UDN visit. We were interested in exploring UDN recommendations related to referrals, treatments, and management changes. We wanted to further understand the experience after a multidisciplinary rare disease visit, whether or not UDN recommendations were the same or different from previous information and recommendations.

## Methods

Wrap-up documents were coded with a deductive approach to understand the UDN multidisciplinary team and the number of UDN evaluations and recommendations provided as part of the visit. Post-evaluation survey data were also coded to characterize participant experiences. Descriptive statistics were used to explore participant and clinical data and survey responses. Finally, statistical models were created to characterize the likelihood of UDN participants receiving recommendations after the UDN visit.

### Data collection

We used two UDN data sources as separate cohorts for this project: 1) wrap-up documents, and 2) post-evaluation surveys to gain the most complete understanding of the UDN participant experience. All participants included in these datasets were evaluated as part of sites involved in the first phase of the UDN project. We extracted all participant demographic and clinical information from the UDN central database, which includes both participant and UDN clinical team-reported information.

#### Wrap-up documents:

At the end of the UDN visit, each UDN clinical site creates a wrap-up document about the participant. The document details visit activities, including advised best next steps (e.g., recommendations and care management changes). Typically, the document includes information about evaluations provided, tests and procedures performed, evaluation results, and integrated recommendations based on collected information. UDN clinical sites provide these documents to participants and upload them to the UDN central database. The UDN site adds an updated wrap-up document if additional information becomes available. We included wrap-up documents written for UDN visits completed between June 2015 and August 2018. 917 participants were evaluated during the study time period; 36 did not have a wrap-up document. Of the 881 wrap-up documents reviewed, 38 were incomplete. As such, the final analysis included 843 wrap-up documents.

- Developing the wrap-up document data collection form: A.N. performed an exploratory inductive review of 20 wrap-up documents (hereafter, “wrap-ups”) to determine the format, content, and terms across sites. Then, A.N. developed a REDCap form to deductively collect and code wrap-up data about evaluations and recommendations. Together, A.N. and L.E.R. applied these results to an initial set of wrap-ups. Finally, A.N. and L.E.R. presented this form to the rest of the team (K.L., A.T.M.) and led a consensus process to finalize wrap-up coding definitions, which included the input of UDN genetic counselors and other site staff.
  - UDN specialist evaluations (hereafter “evaluations”): any specialty department or individual specialist who was part of the participant’s UDN visit either in-person or via telehealth.
  - UDN recommendations: any note made by the UDN that suggested an action be taken by the participant, a family member, or caregiver. Recommendations consistently included four pieces of information: 1) who the recommendation was for (participant, family member/caregiver, or other), 2) recommendation timeline (a recommendation for now, a recommendation to continue something that was already being done, or a conditional recommendation to implement if something changed in the future), 3) type of recommendation: 12 categories - medication, laboratory test, imaging or functional test, medical or surgical procedure, referral to medical provider (whether UDN-affiliated or not), referral to therapy, referral for education or social support, genetic or preconception counseling, referral to other researcher or study, dietary, devices, and lifestyle or behavioral, and 4) whether or not the same recommendation was made by multiple UDN specialists.
- Training coders: Four research assistants (RAs) coded wrap-ups using the REDCap form. L.E.R. trained the research assistants on the coding protocol in an iterative, interactive workshop. The training continued with scaffolded practice opportunities where coders worked together to code six wrap-ups, which were representative of different sites and document lengths. L.E.R. instructed the research assistants to identify any instance of an evaluation or recommendation and select the applicable code in the form. A.N. reviewed each selection and gave detailed feedback individually and collectively to the RAs to increase accuracy. Such consensus discussions helped determine the best way to proceed with coding, including repeating this practice process three times. At this point, RA coding regularly reflected alignment.
- Coding wrap-up documents: After the training period, A.N. randomly assigned RAs to code all wrap-ups, of which A.N. coded 20.0 % (n = 172) to track alignment. Of the wrap-ups reviewed, 80.2 % (138/172) had concordant evaluation coding and 60.5 % (104/172) had concordant recommendation coding. A.N. brought the discrepancy (20 % sample) to the research team for discussion. Together, the team (K.L., A.T.M., A.N., L.E.R.) concluded the data discrepancy almost entirely produced an undercount. Models therefore produced underestimates of the relationships under study.

#### Post-evaluation surveys:

The UDN Coordinating Center administers post-evaluation surveys by phone or email to every UDN participant/caregiver no earlier than 7 days after their visit is complete; most if not all caregivers were at the visit. Team members are accompanied by an interpreter when calling participants who do not speak English, as documented by UDN records. The survey asks questions to gauge UDN participant experience, understanding of visit information, and intended next steps. In this study, we included post-evaluation surveys completed by participants after their UDN visits between December 2016 and June 2019 (n = 396). Central survey data collection began in December 2016. We used survey data about any changes to medications suggested, recommendations made, and participant intention to follow recommendations for this study. Two research assistants independently coded free text responses about medication changes and next steps as “Yes” or “No”. They reviewed any discrepancies and reached consensus on all codes. A research assistant and A.N. coded the participant-reported recommendations based on recommendation codes also used in the wrap-up analysis.

### Data analysis

We used descriptive statistics to explore: 1) participant demographic and clinical data, and 2) survey responses pertaining to recommendations and medication changes. The two datasets were examined separately to take full advantage of available data. Combining the datasets would restrict our understanding of the wrap-up document data to those participants who responded to surveys, a difference of 447 participants (53 % of available data).

Additionally, we created separate models for each dataset (Appendix C) (i.e., wrap-up documents, post-evaluation surveys) to determine characteristics associated with a participant being more or less likely to receive recommendations after the UDN visit (Table 1). The wrap-up document model also included characteristics associated with the number of participant evaluations. In both models, the predictor variables were both *patient-reported (or caregiver-reported)*: age, age of symptom onset, biological sex (participant report of gender not collected), race(a construct relevant because of structural racism), ethnicity, and *UDN-reported*: UDN clinical site, age at evaluation, pediatric or adult (pediatric: <18 years), primary symptom category (as chosen by the UDN site at the time of application review), diagnosis, local/external (‘local’ means that UDN participant was referred by a provider at the UDN-associated medical center; ‘external’ means that the participant was referred from outside the assigned UDN-associated medical center), and whether or not the individual had genetic testing done through the UDN.

Depending on whether the outcome was binary or a count, a Binomial or Poisson regression model was used, respectively. Predictors were selected based on the best available evidence linking the variable to outcome. Due to the number of potential predictors, a 10-fold cross-validated LASSO [Least Absolute Shrinkage and Selection Operator] regression was employed to highlight the outcome predictors most relevant to the question under study [23]. Here, estimates suggest the direction of the relationship but are not interpreted by their effect size.

This project fell under an existing institutional review board (IRB) protocol for the UDN approved by the National Institutes of Health IRB (15-HG-0130). De-identified datasets are provided on request.

## Results

### Demographics and clinical characteristics

Table 2 presents demographic, clinical, and study characteristics for the data sources (i.e., wrap-ups, post-evaluation surveys). Mean participant age at the time of evaluation was 7.8 years for those who were pediatric participants and ranged from 38.4 to 41.2 years for those who were adult participants. Most participants across datasets identified as female, White, and not Hispanic or Latino.

Across datasets, the most common primary symptom category was neurology (wrap-up documents: 46.5 %, post-evaluation survey: 52.0 %). Most participants did not have a “certain” or “highly likely” diagnosis within 3 months of wrap-up document or survey completion (wrap-up documents: 82.0 %, post-evaluation survey: 89.1 %). Across datasets, the majority of UDN participants were external (wrap-up documents: 62.4 %, post-evaluation survey: 68.2 %). The vast majority also had genetic testing as part of their UDN visit (wrap-up documents: 90.5 %, post-evaluation survey: 92.7 %).

### Wrap-up document characteristics

Of the 843 wrap-ups included in the analysis, 771 (91.5 %) included information about evaluations and 836 (99.2 %) had information about recommendations.

#### UDN Evaluations:

The mean number of evaluations per participant was 3.8 (median: 3.0). The number of evaluations ranged from 0 to 18. All patients were evaluated by at least one specialist. Zero here indicates information about the number of specialists was not documented. Of the 771 wrap-ups with evaluation information, the most common evaluation was Genetics, with over 68.7 % of participants having been evaluated by a genetics specialist. Other common evaluations were Neurology (47.3 %) and Ophthalmology or Neuro-ophthalmology (44.0 %).

#### UDN Recommendations:

The mean number of recommendations per participant was 4.9 (median: 3.0). Of the 836 wrap-ups with recommendations, 681 (81.5 %) had ≥ 1 recommendation. The number of recommendations per participant ranged from 0 to 42. The most common type of recommendation was referral to a medical provider (452 (54.1 %) had ≥ 1). The next most common recommendation categories were medication changes (353 (42.2 %) had ≥ 1) and follow-up for imaging or functional studies (272 (32.5 %) had ≥ 1).

### Post-evaluation survey characteristics

Of 396 patient surveys completed, 376 surveys had responses for the medication changes question. Of those, 89 (23.6 %) noted medication changes were recommended at the end of the UDN visit. 347 surveys had responses for the recommendations question. Of those, 192 (55.3 %) had recommendations made for next steps. The most common recommendation was referral to a medical provider (63 (32.8 %)). The second most common recommendation was for a test or procedure (37 (19.2 %)). Of the 192 participants who noted recommendations, 101 (52.6 %) said they intended to follow the recommendations.

### Statistical modeling

Table 3 shows variables identified by the models. Post-evaluation survey models P1 and P2 did not yield any statistically significant variables. All other models identified clinical site, age at evaluation, primary symptom category, and local/external status of the participant as variables related to evaluations, recommendations, or medication changes, and were therefore included in the model.

Table 4 presents the Binomial and Poisson regression model results. Only variables with p-values of < 0.05 are reported here. All estimates are in comparison to the reference variables, chosen because they are the most populous categories for comparison (Clinical Site: 1, Primary Symptom Category: neurology, Race: White).

### UDN evaluation count (Model W1)

Older age at evaluation and being an external participant were associated with fewer specialist evaluations as part of the UDN visit (estimate: −0.077, p-value: 0.001). Having a diagnosis within 3 months of the UDN visit (estimate: 0.06, p-value: 0.03) and having a primary symptom category of allergies and immunology or rheumatology were associated with more evaluations (estimate: 0.22 and 0.27, p-value: 0.027 and 0.032, respectively). Various sites were associated with higher or lower evaluation counts.

### UDN recommendation count (Model W2)

Older age at evaluation (estimate: −0.008, p-value: <0.001), being an external participant (estimate: −0.138, p-value: <0.001), female sex (estimate: −0.048, p-value: <0.001), having a diagnosis (estimate: −0.08, p-value: 0.001), and reporting multiple races (estimate: −1.544, p-value: 0.012) were associated with fewer recommendations. Having a primary symptom category of gynecology (estimate: 0.223, p-value: 0.014), rheumatology (estimate: 0.567, p-value: <0.001), or urology (estimate: 0.23, p-value: 0.033) and reporting race as Asian (estimate: 0.303, p-value: 0.012), Black or African American (estimate: 0.271, p-value: 0.044), or American Indian or Alaskan Native (estimate: 0.269, p-value: 0.042) were associated with more recommendations. There were site-specific differences in the recommendation counts, with some sites having greater or fewer recommendations than the comparison site

### Changes to medications made by the UDN (yes/no) (Model P3)

Overall, being an external participant (estimate: −0.44, p-value: 0.02) was associated with having fewer medication changes suggested by the UDN; one site (estimate: −1.434, p-value: 0.01) was also associated with fewer medication changes.

## Discussion

To date, outcomes of UDN multidisciplinary visits that generally include genomic testing have not been extensively studied related to medical recommendations for changes in care. Therefore, we sought to: 1) explore recommendations made as part of the UDN visit as well as the number and type of specialists involved and 2) identify factors that led to recommended changes in care following the UDN visit. In doing so, we identified UDN evaluation and recommendation patterns as well as characteristics concerning their frequency as part of a rare disease multidisciplinary evaluation.

### Recommendations made by the UDN

The wrap-up analysis highlights that the vast majority of UDN participants (81.5 %) received one or more recommendations from their UDN site following their visit. This suggests actionable information coming from the visit that has the potential to impact downstream care. On the participant-reported post-evaluation surveys, most respondents also noted recommendations were made by the UDN site (55.3 %); however, the percentage was lower than that in the wrap-up document analysis. Although these are discrete datasets, and comparability is limited, the results nonetheless suggest a possible discrepancy in what is considered a recommendation by UDN clinicians and researchers versus participants and caregivers. There may also be a lack of, or delay in, communication about recommendations, such that information delivered to participants while at the visit may differ from what is written down in a wrap-up document, or be differently or not at all recalled by the participant. Likewise, clinical study sites might think recommendations have been clearly conveyed, when in fact they have not. Further investigation is warranted; making an informed decision about whether to follow through with recommended changes in care depends on providers and institutions giving participants and/or caregivers accessible, understandable, and actionable information and tasks [24-26].

### Participant intent-to-follow recommendations

In the survey, over half of participants reported their intent to follow recommendations made by the UDN. Specifically, it seems most participants noted they intend to follow through with suggested changes in care and may need further consultation to do so.

### Factors associated with the number of UDN evaluations and recommendations

Receiving a diagnosis within 3 months of the UDN visit was shown to be associated with a higher number of UDN specialist evaluations as recorded in wrap-ups (Model W1). Although we cannot determine causality, input from more specialists (i.e., higher number of evaluations) during the multidisciplinary visit may be more likely to yield a diagnosis. Other studies have found that multidisciplinary approaches lead to changes in diagnosis and higher than expected diagnostic rates [27-29]. Furthermore, a study by Barnett et. al revealed that groups of physicians had higher diagnostic accuracy than individual physicians, and that groups outperformed individual specialists in their subspecialty [30]. The association between number of specialist evaluations, specialist collaboration, and likelihood of diagnosis warrants further exploration.

Having a diagnosis was also associated with fewer recommendations recorded in wrap-ups (Model W2). One hypothesis is that input from more specialists (i.e., more evaluations) leads to a potential diagnosis, in turn leading to fewer but likely more specifically tailored recommendations. It also highlights that, even without a diagnosis, participants in the UDN are likely to receive recommendations regarding their subsequent care. Prior UDN pediatric and adult focus group analysis demonstrated that participation in the multidisciplinary UDN visit was valuable, whether or not a diagnosis was reached [22].

In addition, older age at evaluation was associated with fewer UDN specialist evaluations and recommendations (Models W1, W2). This may be because fewer options exist for adults with medical complexity [31,32]. In focus group analysis, we saw that UDN adult participants specifically note the lack of support and careful evaluation by medical providers outside of the UDN context [22].

On the whole, race was associated with more recommendations of any type. (Model W2). The research literature generally demonstrates that race is associated with less health access and care [33-36], due to structural barriers. This is also seen in the setting of rare genetic disease [37,38]. Therefore, we may be seeing the effect of this when patients with previously inadequate access and treatment encounter specialized, multidisciplinary care, such as the UDN. However, participants who identified as “multiple race” received fewer recommendations. Further investigation is warranted to explore the role of structural barriers and participant experience.

Across datasets, we saw notable differences in both evaluation and recommendation counts by site (Models W1, W2, P3,) and primary symptom (Models W1, W2). These differences may suggest varied practices at the sites themselves or across specialties, such as different thresholds for scheduling evaluations or making recommendations. They could also be related to different sites or specialist practices for writing wrap-ups. Regardless, these discrepancies warrant further exploration to identify the underlying cause.

### Future directions

From these analyses we see that after UDN multidisciplinary visits a variety of recommendations are made (e.g., increase in services, care management), regardless of whether they are new or not. This is true for participants who receive a diagnosis as well as for those who do not, which is consistent with prior analyses [18]. Even so, discrepancies are evident in that UDN specialists, participants, and families may be interpreting the meaning of a recommendation differently. Perhaps too, UDN communications about recommendations and next steps are not always clear. Given that participant and caregiver recommendation follow-up is crucial to changes in care, additional investigation of recommendation communication is needed. In addition, while we were able to assess recommendations and intent to follow them, data collection surrounding changes in care and symptoms over time was not available. Best practice recommendations could be developed for writing (i.e., standardized process across sites) and using (i.e., better assessment of follow-up communication, care and outcomes over time) wrap-up documents. Additional areas of focus for future research and practice include site discrepancies in reported evaluations and recommendations, communication over time related to care management, and participant characteristics related to structural barriers (e.g., race, gender, age) that may be associated with the frequency of evaluations and recommendations, signaling systemic barriers in care.

### Limitations

This study had several limitations. First, we were limited in drawing conclusions that involved comparisons across the two samples, because there wasn’t complete participant overlap. Based on these data, it is also unclear whether participant-reported recommendations were only those offered at the time of the UDN visit and/or from reading wrap-up documents, after the visit, or provided by clinicians outside the UDN, whether prior to or after the visit. Additionally, there may be some survey response bias; perhaps respondents felt pressured to say they were following UDN recommendations or patients with certain clinical characteristics (e.g., more symptoms) were less likely to respond. Finally, several demographic categories had small sample sizes (e.g., Native Hawaiian, American Indian) which restricted a more complex stratified analysis to understand the UDN experience across the study population.

## Conclusions

UDN multidisciplinary visits involve multiple evaluations by many specialists, resulting in numerous recommendations. The collaborative approach to assessing the whole patient is largely different from prior care received in genetics clinics or individual specialist offices. Future research will help us better understand how to maximize the UDN, and other multidisciplinary, visit experiences and communicate about ongoing care management. The collaborative team consultation approach can be optimized to harness the powerful evaluations and recommendations garnered from a UDN visit.

## Supplementary Material

1

2

3

## Figures and Tables

**Table 1:** Analytic models.

Dataset	Model	Outcome Variable	Model Type	Description
Wrap-up documents (provider-reported)	W1	UDN evaluation count	Poisson	Analyzed characteristics of UDN participants associated with having more specialist evaluations during the UDN visit based on information provided in wrap-up documents
	W2	UDN recommendation count	Poisson	Analyzed characteristics of UDN participants associated with having more recommendations made by UDN specialists based on information provided in wrap-up documents
Post-evaluation surveys (participant-reported)	P1	UDN recommendations made (y/n)	Binomial	Analyzed characteristics of UDN participants associated with having recommendations made as reported by participants in post-evaluation surveys
	P2	UDN recommendation count	Poisson	Analyzed characteristics of UDN participants associated with having more recommendations made by UDN specialists as reported by participants in post-evaluation surveys
	P3	Changes to medications made by the UDN (y/n)	Binomial	Analyzed characteristics of UDN participants associated with medication changes being suggested by UDN specialists as reported by participants in post-evaluation surveys

Table 1 describes the datasets, the outcome variables, the model types, and a description for each model.

**Table 2:** Demographic and clinical characteristics.

	Wrap-up Documents	Post Evaluation Survey
	Total (N = 843)	Total (N = 396)
**Age at evaluation** ^i^		
Pediatric		
Mean (SD)	7.8 (4.7)	7.8 (4.6)
Median (Min, Max)	6.8 (0.2, 18.0)	6.5 (0.7, 17.9)
Adult		
Mean (SD)	38.4 (15.3)	41.2 (15.8)
Median (Min, Max)	35.6 (18.0, 77.3)	38.4 (18.0, 77.3)
**Age of symptom onset** ^ii^		
Pediatric		
Mean (SD)	1.7 (3.2)	1.9 (3.4)
Median (Min, Max)	0.3 (0.0, 15.9)	0.4 (0.0, 15.9)
Adult		
Mean (SD)	24.0 (19.0)	26.4 (20.6)
Median (Min, Max)	21.3 (0.0, 75.5)	24.6 (0.0, 75.5)
	n (%)	n (%)
**Sex** ^ii^		
Female	439 (52.1 %)	199 (50.3 %)
Male	404 (47.9 %)	197 (49.7 %)
**Race** ^ii^		
White	689 (81.7 %)	330 (83.3 %)
Asian	50 (5.9 %)	25 (6.3 %)
Black or African American	44 (5.2 %)	22 (5.6 %)
American Indian or Alaska Native	2 (0.2 %)	0 (0.0 %)
Native Hawaiian or Pacific Islander	1 (0.1 %)	0 (0.0 %)
Multiple races	39 (4.6 %)	15 (3.8 %)
Not Reported	18 (2.1 %)	4 (1.0 %)
**Ethnicity** ^ii^		
Not Hispanic or Latino	618 (73.3 %)	312 (78.8 %)
Hispanic or Latino	125 (14.8 %)	41 (10.4 %)
Unknown or Not Reported	100 (11.9 %)	43 (10.9 %)
**Primary Symptom Category** ^i^		
Allergies and disorders of the immune system	45 (5.3 %)	13 (3.3 %)
Cardiology and vascular conditions	26 (3.1 %)	13 (3.3 %)
Dermatology	6 (0.7 %)	1 (0.3 %)
Endocrinology	18 (2.1 %)	11 (2.8 %)
Gastroenterology	27 (3.2 %)	10 (2.5 %)
Gynecology and reproductive medicine	1 (0.1 %)	1 (0.3 %)
Hematology	7 (0.8 %)	8 (2.0 %)
Infectious diseases	7 (0.8 %)	2 (0.5 %)
Multiple Congenital Anomalies	**93 (11.0 %)**	**31 (7.8 %)**
Musculoskeletal and orthopedics	**69 (8.2 %)**	**35 (8.8 %)**
Nephrology	10 (1.2 %)	3 (0.8 %)
Neurology	**392 (46.5 %)**	**206 (52.0 %)**
Oncology	2 (0.2 %)	1 (0.3 %)
Ophthalmology	4 (0.5 %)	2 (0.5 %)
Other	44 (5.2 %)	17 (4.3 %)
Psychiatry	2 (0.2 %)	1 (0.3 %)
Pulmonology	13 (1.5 %)	3 (0.8 %)
Rheumatology	23 (2.7 %)	13 (3.3 %)
Urology	1 (0.1 %)	0 (0.0 %)
N/A	53 (6.3 %)	25 (6.3 %)
**Diagnosis** ^i^		
Undiagnosed after evaluation	691 (82.0 %)	353 (89.1 %)
Diagnosed after evaluation	152 (18.0 %)	43 (10.9 %)
**Local/External Participant** ^i^		
External	526 (62.4 %)	270 (68.2 %)
Local	317 (37.6 %)	126 (31.8 %)
**Genetic testing** ^i^		
Genetic testing performed during evaluation	763 (90.5 %)	367 (92.7 %)
Genetic testing not performed during evaluation	80 (9.5 %)	29 (7.3 %)
**Survey Respondent** ^i^		
Participant (or participant with family member)	N/A	109 (27.5 %)
Other Relative (or spouse)	N/A	286 (72.2 %)
Missing Information	N/A	1 (0.3 %)
**Clinical Site** ^i^		
Site 1	**237 (28.1 %)**	**121 (30.6 %)**
Site 2	**134 (15.9 %)**	41 (10.4 %)
Site 3	**121 (14.2 %)**	**49 (12.4 %)**
Site 4	84 (10.0 %)	46 (11.6 %)
Site 5	81 (9.6 %)	44 (11.1 %)
Site 6	106 (12.6 %)	**49 (12.4 %)**
Site 7	80 (9.5 %)	46 (11.6 %)

Table 2 describes the demographic and clinical characteristics of both datasets used in the study.

i Provider-reported

ii Participant-reported

**Table 3:** Variables chosen by LASSO for each model.

Variables	Wrap-up documents		Post-evaluation surveys		
		
	W1	W2	P1	P2	P3
**Age at evaluation** ^i^	X	X			X
**Age of symptom onset** ^ii^	*Not included*	*Not included*			X
**Sex** ^ii^		X			
**Race** ^ii^	X	X			
**Ethnicity** ^ii^	X				
**Primary symptom category** ^i^	X	X			X
**Diagnosis** ^i^	X	X			
**Local or external** ^i^	X	X			X
**Genetic testing** ^i^					
**Clinical site** ^i^	X	X			X

Table 3 describes the study variables from each dataset and what the LASSO analysis chose for each model.

i Provider-reported

ii Participant-reported

**Table 4:** Results by model.

Model*	Variable	Estimate	Std. Error	P-value
***Wrap-up documents (provider-reported)***				
W1 - UDN evaluation count	Age at evaluation	−0.009	0.001	< 0.001
	External	−0.077	0.024	0.001
	Primary symptom - Allergies and Immunology	0.224	0.101	0.027
	Primary symptom - Rheumatology	0.27	0.126	0.032
	Diagnosis	0.06	0.028	0.029
	Site #2	0.421	0.045	< 0.001
	Site #3	0.605	0.044	< 0.001
	Site #6	−0.555	0.094	< 0.001
	Site #7	−0.281	0.062	< 0.001
W2 - UDN recommendation count	Age at evaluation	−0.008	0.001	< 0.001
	External	−0.138	0.022	< 0.001
	Primary symptom - Gynecology	0.223	0.09	0.014
	Primary symptom - Rheumatology	0.567	0.129	< 0.001
	Primary symptom - Urology	0.23	0.108	0.033
	Diagnosis	−0.08	0.024	0.001
	Female Sex	−0.048	0.016	0.003
	Race - Asian	0.303	0.121	0.012
	Race - Black or African American	0.271	0.134	0.044
	Race - American Indian or Alaskan Native	0.269	0.132	0.042
	Race - Multiple Races	−1.544	0.612	0.012
	Race - Not reported	0.411	0.136	0.003
	Site #2	0.842	0.038	< 0.001
	Site #3	0.632	0.039	< 0.001
	Site #4	−0.276	0.061	< 0.001
	Site #6	−0.628	0.078	< 0.001
	Site #7	−0.684	0.065	< 0.001
***Post-evaluation surveys (participant-reported)***				
P3 - Changes to medications made by the UDN (y/n)	External	−0.435	0.188	0.021
	Site #5	−1.434	0.559	0.010

Table 4 describes the results for each model by dataset.

* For models P1 and P2, no variables were chosen by LASSO, so no results are shown.

## Data Availability

Data will be made available on request.

## Appendix A. Supporting information

Supplementary data associated with this article can be found in the online version at doi:10.1016/j.rare.2025.100105.
