# Supplementary material for: Insights from multidisciplinary rare disease visits: Findings from wrap-up documents and participant surveys in a national diagnostic study
Source: Rare. Author manuscript; Available in PMC 2025 Dec 23. (PMC12721797; doi:10.1016/j.rare.2025.100105)
Supplement: 1 [file NIHMS2127932-supplement-1.docx]

Post-evaluation survey questions

1. Were changes to the medications suggested at the end of the UDN visit? If so, what were they and do you intend to follow the recommendations?
2. Were recommendations made for next steps (e.g. additional testing, referrals, etc.)? If so, what were they and do you intend to follow the recommendations?
