# Supplementary material for: Insights from multidisciplinary rare disease visits: Findings from wrap-up documents and participant surveys in a national diagnostic study
Source: Rare. Author manuscript; Available in PMC 2025 Dec 23. (PMC12721797; doi:10.1016/j.rare.2025.100105)
Supplement: 2 [file NIHMS2127932-supplement-2.docx]

# Wrap up Info and Evaluation Count

Record ID

Evaluations
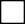
 Audiology


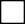
 Cardiology
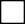
 Dentistry
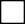
 Dermatology


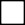
 Developmental/Neurodevelopmental Medicine
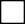
 Endocrinology


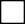
 Gastroenterology
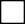
 Genetics


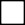
 Gynecology
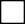
 Hematology
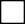
 Hepatology


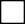
 Immunology / Allergy-Immunology
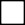
 Infectious Disease


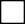
 Nephrology
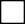
 Neurology


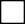
 Neuropsychology / Neurocognitive
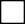
 Nutrition


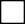
 Oncology


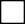
 Ophthalmology/Neuro-ophthalmology
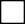
 Orthopedics


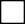
 Otolaryngology
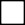
 Pain Management
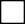
 Palliative Care
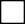
 Pathology


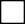
 Pediatrics


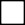
 Physical Medicine/PT/OT/Speech Therapy
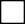
 Plastic surgery (including Craniofacial)
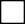
 Pulmonology


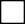
 Psychiatry / Psychology
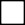
 Radiology


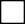
 Rheumatology
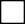
 Surgery


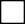
 Urology
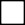
 Other
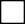
 Other 2


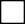
 Other 3


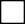
 Other 4


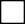
 Other 5


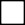
 No information about evaluations present
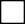
 Participant was not evaluated

Please describe Other:

Please describe Other 2:

Please describe Other 3:

Please describe Other 4:

Please describe Other 5:

Were recommendations made? No recommendations

No information present Recommendation(s) present


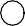

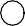

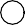


(If recommendations present, please fill out a recommendation form for each)

Notes about Wrap-up document

# Recommendation Count

Person whose care will change based on recommendation Proband

Family member or caregiver Other


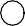

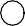

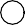


Please specify "other":

Recommendation timeline Recommendation for now Recommendation to continue Recommendation for future

Recommendation action Start / Do action

Stop Change


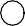

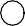

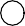

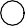

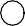

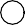

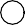

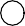

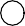


Avoid / Negative recommendation Unknown

Other

Please specify "other":

Type of Recommendation Medication

Laboratory Test


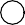

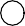

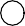

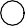

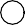

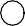

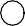

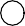

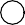

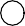

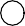

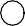

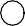

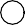


Imaging or Functional Test Medical or surgical procedure Referral to medical provider Referral for therapy

Referral for education or social support Genetic or preconception counseling Referral to other researcher or study Dietary

Devices

Lifestyle and Behavioral

Not specified/not enough details Other

Please select referral specialty Audiology Cardiology Dentistry Dermatology


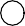

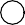

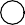

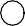

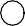

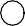

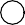

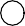

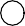

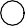

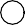

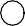

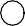

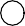

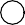

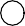

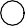

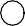

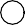

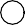

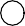

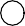

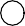

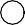

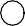

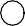

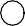

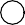

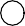

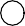

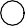


Developmental/Neurodevelopmental Medicine Endocrinology

Gastroenterology Genetics Gynecology Hematology Hepatology

Immunology / Allergy-Immunology Infectious Disease

Nephrology Neurology

Neuropsychology / Neurocognitive Nutrition

Oncology

Ophthalmology/Neuro-ophthalmology Orthopedics

Otolaryngology Pain Management Palliative Care Pathology Pediatrics

Physical Medicine/PT/OT/Speech Therapy Plastic surgery (including Craniofacial) Primary Care

Pulmonology

Psychiatry / Psychology Radiology

Rheumatology Surgery Urology Other

Unclear / No information

Please describe "other":

Please describe "other"

Type of Therapy Physical therapy

Speech therapy

Occupational therapy

Mental health counseling therapy Behavioral therapy

Other

Please specify "other":

Type of Referral for educational or social support IEP/504 or other educational support

Support or advocacy group Other

Please specify "other":

*Page 5*

Please describe lifestyle and behavioral:

Was this recommendation made by multiple providers? Yes

No

How many providers made this recommendation?

Brief description of recommendation

(Ex. type of test (MRI, CT, etc.), name of medication, name of procedure)

Notes about Recommendation

(Please include direct quote and short rationale)

Please check if you would like this recommendation to Yes be reviewed.
