# Supplementary material for: Insights from multidisciplinary rare disease visits: Findings from wrap-up documents and participant surveys in a national diagnostic study
Source: Rare. Author manuscript; Available in PMC 2025 Dec 23. (PMC12721797; doi:10.1016/j.rare.2025.100105)
Supplement: 3 [file NIHMS2127932-supplement-3.pdf]

Appendix C: LASSO, Binomial and Poisson Statistical Models

Statistical models used to explore the UDN multidisciplinary team visit.

(1) Binomial (logistic) regression

$$Y_i \sim \text{Bernoulli}\big(\pi_i\big), \quad i = 1, \ldots, n,$$
$$\text{logit}\big(\pi_i\big) = \beta_0 + \sum_{j=1}^{p} x_{ij}\,\beta_j. \tag{1}$$

(2) Poisson regression (count of post-visit evaluations)

$$Y_i \sim \text{Poisson}\big(\mu_i\big),$$
$$\log\big(\mu_i\big) = \beta_0 + \sum_{j=1}^{p} x_{ij}\,\beta_j, \tag{2}$$

(3) LASSO-penalised maximum-likelihood estimator

$$\hat{\boldsymbol{\beta}} \;=\; \arg\min_{\boldsymbol{\beta}} \Big\{ -\ell(\boldsymbol{\beta}) + \lambda \sum_{j=1}^{p} |\beta_j| \Big\}, \tag{3}$$

where $\ell(\boldsymbol{\beta})$ is the average log-likelihood of model (1) or (2), and λ is the tuning parameter selected by 10-fold cross-validation. The intercept β_0 is left unpenalised.
